# The role of digital health in growth hormone therapy: perspectives from Gulf Cooperation Council pediatric endocrinologists

**DOI:** 10.3389/fendo.2025.1641513

**Published:** 2025-10-15

**Authors:** Walid Kaplan, Abdullah Alherbish, Abdullah Aljnaibi, Afaf Alsagheir, Angham Almutair, Aqeel Farooque, Asma Deeb, Bassam Bin Abbas, Jamal Al Jubeh, Najya Attia, Nandu Thalange, Sareea Salem Al Remeithi, Paul Dimitri, Octavio Rivera-Romero, Ekaterina Koledova

**Affiliations:** ^1^ Department of Pediatrics, Tawam Hospital, AI Ain, United Arab Emirates; ^2^ Al Habib Medical Group, Riyadh, Saudi Arabia; ^3^ Danat Al Emarat Hospital, Abu Dhabi, United Arab Emirates; ^4^ Department of Pediatric Endocrinology, King Faisal Specialist Hospital and Research Center, Riyadh, Saudi Arabia; ^5^ Department of Pediatric, College of Medicine, King Saud bin Abdulaziz University for Health Sciences, King Abdullah International Medical Research Center, and King Abdullah Specialist Children’s Hospital, Ministry of National Guard Health Affairs (MNGHA), Riyadh, Saudi Arabia; ^6^ Department of Pediatrics, Al Qassimi Hospital, Sharjah, United Arab Emirates; ^7^ Sheikh Shakhbout Medical City and College of Medicine and Health Sciences, Khalifa University, Abu Dhabi, United Arab Emirates; ^8^ Gulf Medical School, Ajman, United Arab Emirates; ^9^ Department of Pediatrics Endocrinology, King Faisal Specialist Hospital and Research Center, Riyadh, Saudi Arabia; ^10^ Sheikh Khalifa Medical City, Abu Dhabi, United Arab Emirates; ^11^ King Saud bin Abdulaziz University for Health Sciences, Jeddah King Abdullah Specialized Children’s Hospital (KASCH), Jeddah, Saudi Arabia; ^12^ Al Jalila Children’s Specialty Hospital, Dubai, United Arab Emirates; ^13^ Department of Pediatrics, Sheikh Khalifa Medical City, Division of Endocrinology, Abu Dhabi, United Arab Emirates; ^14^ The Department of Pediatric Endocrinology, Sheffield Children’s NHS Foundation Trust, Sheffield, United Kingdom; ^15^ Electronic Technology Department, Universidad de Sevilla, Seville, Spain; ^16^ Global Medical Affairs, Cardiometabolic and Endocrinology, Merck Healthcare KGaA, Darmstadt, Germany

**Keywords:** digital health, growth hormone treatment, pediatric endocrinology, adherence, monitoring

## Abstract

**Background:**

With the increasing use of digital health tools patient-generated health data play a crucial role in clinical decision-making, particularly for monitoring treatment adherence. However, integrating data into routine practice remains challenging, especially for chronic conditions such as growth disorders requiring growth hormone therapy (GHT). Integrating these data is essential to improve treatment adherence and growth outcomes in pediatric patients on GHT.

**Aim:**

To explore perspectives of pediatric endocrinologists in the Gulf Cooperation Council (GCC) region on patient-generated health data for improving GHT adherence and identified strategies for integrating such data into clinical practice.

**Methods:**

A participatory workshop was conducted on March 2, 2024, in Dubai, United Arab Emirates, using the nominal group technique. Twelve pediatric endocrinologists from the GCC region, one chairman, and two moderators participated in the session. The session centered on three clinical scenarios: GHT naïve (recently diagnosed), poorly adherent, and poor responders. Through two structured voting rounds, experts individually identified, discussed, and ranked the top five most relevant and useful patient-generated health data factors. The first round prioritized key factors, while the second round allowed participants to reassess and refine their selections to reach consensus. The final discussion focused on how identified factors could integrate into clinical practice.

**Results:**

Twenty-two influencing factors were identified, representing the most relevant and useful types of patient-generated health data for integration into clinical practice. Top factors in the first ranking round included demographic data (21 points: age, income level, familiarity with technology); patient’s feelings about treatments and satisfaction (19 points); and social background (17 points: family support, insurance, caregiving responsibilities). Other considerations included reasons for missed injections and educational needs (15 points each). In the second round, social background (35 points) ranked highest, followed by injection context (34 points: timing, comfort, administration support) and patient’s feelings about treatments and satisfaction (30 points) emphasized motivational and emotional aspects of adherence.

**Conclusion:**

The study highlights the significant role of social background, injection contexts, and patient satisfaction as key patient-generated health data factors for pediatric endocrinologists in the GCC region. These findings highlight their potential integration into GHT workflows to enhance clinical decision-making.

## 1 Introduction

Growth hormone deficiency (GHD) in children is a chronic endocrine disorder that leads to growth retardation and various metabolic abnormalities (1, 2). The global prevalence of pediatric GHD in children is estimated at 1 in 4,000–10,000 live births, with higher detection rates in developed healthcare systems (3). Standard treatment involves subcutaneous injections of recombinant human growth hormone (r-hGH), to improve growth velocity and metabolic health outcomes (4, 5). However, long-term adherence to growth hormone therapy (GHT) remains a global challenge, with adherence rates ranging from 73% to 95% within 12 months of treatment initiation and often declining thereafter (6).

Suboptimal adherence to GHT compromises growth outcomes and increase metabolic risks and healthcare costs (7). Contributing factors to poor adherence include injection burden, discomfort, psychological stress, and insufficient caregiver supervision (8). Real-world data from Latin America showed that children with high adherence achieved a mean catch-up growth of +0.69 standard deviation (SD) over 24 months compared with +0.52 SD in children with low or intermediate adherence (9). Emerging evidence suggests that connected injection devices can support catch-up growth in children with growth disorders, highlighting their clinical utility (10).

Remote patient monitoring is a promising approach to address adherence barriers and optimize treatment efficacy (11). Patient-generated health data collected using mobile health applications, wearable devices, and smart medical tools can support treatment adherence by enabling monitoring of injection schedules, tracking of symptoms, and reinforcement of healthy lifestyle habits (12, 13). Healthcare professionals (HCPs) play a crucial role in recommending and facilitating the adoption of these digital health solutions, directly influencing caregiver engagement and treatment persistence (14).

Smart injection devices, such as Easypod^®^ combined with the Growzen^®^ application, enable real-time tracking of injection adherence, and timely intervention when adherence declines (15). The coronavirus disease pandemic in 2019 accelerated the adoption of telemedicine and patient-generated health data in the management of pediatric endocrine disorders (16). However, the integration of patient-generated health data into routine clinical practice remains challenging due to concerns regarding data accuracy, uptake of digital solutions, and interoperability with electronic health records (EHRs) (17, 18).

The use of digital health solutions for pediatric endocrine disorders has grown significantly with substantial investment in e-health infrastructure, telemedicine, and precision medicine to improve pediatric healthcare across the Gulf Cooperation Council (GCC) region (17). These digital health solutions enable HCPs to monitor and treat patients remotely, provide valuable insights, and facilitate the collection of real-world data, while also helping to personalize and improve treatment outcomes (11, 14). However, regional data regarding the use of patient-generated health data in GHT remain limited.

This study aimed to explore the perspectives of pediatric endocrinologists in the GCC region on the most relevant types of patient-generated health data collected through digital health solutions to improve adherence to GHT. It also examined challenges in integrating these data into clinical practice and explored strategies to enhance their use and adoption across the region. The identified factors may help in day to day clinical decision making, strengthen physician-caregiver discussions, and support practical integration into GHT workflows.

## 2 Methods

### 2.1 Study design

A participatory workshop utilizing the nominal group technique (NGT) was conducted on March 02, 2024, in Dubai, United Arab Emirates (UAE), to establish expert agreement on optimizing GHT outcomes in the GCC region through patient-generated health data utilization. The session incorporated structured NGT methodology to ensure systematic data collection and evaluation through defined discussion phases and voting rounds.

### 2.2 Participants

The workshop included 12 senior pediatric endocrinologists from the GCC countries (UAE and Saudi Arabia), all with over 15 years of experience managing pediatric growth disorders, and expertise in both patient-generated health data and digital health solutions. All participants were from diverse healthcare settings, primarily public and semi-private hospitals, typically managing more than 100 patients on GH therapy. Two digital health specialists with extensive experience in consensus methodologies moderated the discussions. The role of moderators was to facilitate balanced participation, ensure all the viewpoints were heard, and guide the group through each structured phase of the NGT process without influencing the content of the discussion. The session was chaired by an expert pediatric endocrinologist with 21 years of experience and a particular interest in health technology solutions to advance pediatric healthcare. The chair provided overall guidance and validation of the final ranked factors.

### 2.3 Scenarios used in the NGT process

Three clinically relevant patient scenarios were used to guide and facilitate discussions and about the challenges and opportunities of integrating patient-generated health data to contextualize the responses. These scenarios were selected to reflect real-world clinical scenarios relating to patient adherence and treatment response during GHT:

Scenario 1: GH naïve (recently diagnosed)

- This group represented children newly diagnosed with GHD and not yet prescribed therapy.
- Experts discussed which types of patient-generated health data were most relevant to support treatment initiation, guide maintenance and serve as a tool to educate caregivers and patients.

Scenario 2: Poorly adherent

- This group represented patients not following the recommended GHT dose schedule.
- Experts discussed how patient-generated health data could help track adherence and identify barriers (e.g., injection anxiety and forgetfulness).

Scenario 3: Poor responders

- This group included patients who adhered to the prescribed GH treatment but did not achieve the expected growth outcomes.
- Experts discussed how patient-generated health data could help understand the patient’s context and guide therapy adjustments.

### 2.4 Nominal group technique process and data collection

**Figure 1:**
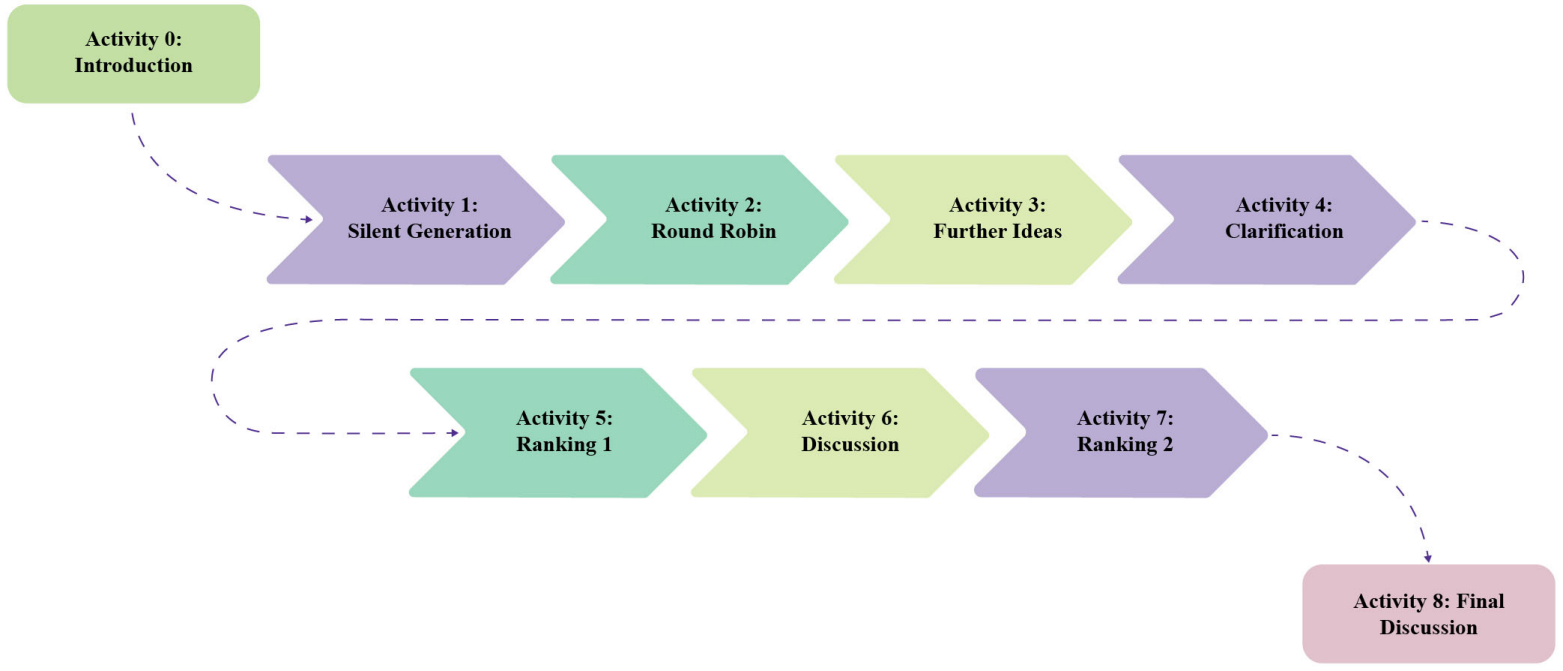
NGT session structure used in the expert workshop. Activity 0 – Introduction (5 min): Moderators explained the objective and workshop process; Activity 1 - Silent Generation (15 min): Experts independently listed factors influencing patient-generated health data integration; Activity 2 - Round Robin (20 min): Each participant shared one factor in turn; Activity 3: Further ideas: Additional factors were contributed after listening to peers; Activity 4 – Clarification (30 min): Similar ideas were discussed and grouped thematically; Activity 5 - Ranking 1 (30 min): Experts ranked their top five factors individually; Activity 6 – Discussion (10 min): Moderators led a group discussion on Ranking outcomes; Activity 7 - Ranking 2 (15 min): Experts re-ranked factors based on the discussion; Activity 8 - Final discussion (10 min): Consensus was reached on priority factors for patient-generated health data integration. NGT, nominal group technique.

A systematic prioritization of patient-generated health data was conducted using an eight-step structured NGT approach (**Figure 1**). Experts initially identified relevant factors independently, followed by structured sharing and refinements based on group suggestions. The process included clarification and thematic grouping of similar items prior to ranking.

For quantitative prioritization, a weighted scoring system was used: first-ranked factors received 5 points, second-ranked: 4 points, third-ranked: 3 points, fourth-ranked: 2 points, and fifth-ranked: 1 point. Only factors selected by at least four participants proceeded to a second round of voting for final prioritization and consensus. The total score for each factor was calculated by aggregating individual expert rankings, ensuring data-driven prioritization.

## 3 Results

The NGT session identified and prioritized key patient-generated health data factors that may influence GHT adherence in the GCC region. The findings included the initial list of factors, the ranking process, expert consensus, and the factors prioritized for clinical implementation.

### 3.1 Identified factors

The expert panel initially proposed 14 potential factors that they believed influence the integration of patient-generated health data to monitor and support GHT adherence. Through iterative discussions and refinement, 8 additional factors were incorporated, resulting in a final set of 22 clinically relevant defined factors (explanation of these factors are detailed in **Table 1**). For analytical clarity, these factors were organized under thematic categories based on conceptual similarity. Notably, during the expert discussion, it was agreed that factor 21 (patient satisfaction*)*, and factor 10 (patient’s feelings about treatment*)* were conceptually merged into single factor as the expert group reached consensus that patient satisfaction is an intrinsic component of the overall patient experience with therapy. This merged factor was used for the ranking process.

**Table 1:** Key factors influencing patient-generated health data integration in GHT.

Category	Factor	Description
Treatment-related	Other treatment/medication (F1)	Conflicts with other therapies affecting adherence
	Injection contexts* (F2)	Location, comfort level, timing of administration, and assistance in administering injections
	Reason for missing injections^#^ (F3)	Forgetfulness, avoidance, or side effects
Patient-specific	Social background (F4)	Family structure and cultural influences
	Psychological background (F5)	Anxiety, stress, or emotional concerns
	Patient’s expectations (F6)	Expected treatment outcomes and adherence perception
External influences	Other conditions/illnesses (F7)	Comorbidities affecting GHT adherence
	Data from other patients (local) (F8)	Influence of shared experience on treatment decisions
Practical barriers	Missed injections (F9)	Frequency and reasons behind missed doses
	Patient’s feelings about treatment (F10)	Feeling about treatment as a treatment, burden or expectation, challenges faced, feelings about results and expectations
	Complications/Side effects (F11)	Symptoms affecting adherence
Demographic and lifestyle	Demographic data (F12)	Age, income, and familiarity with technology
	Quality of life^$^ (F13)	Measured using validated questionnaires
	Education needs (F14)	Information gaps affecting adherence
	Patient experience with doctors (F15)	Perceived quality of medical treatment
	Sleep patterns^^^ (F16)	Patient’s sleep behavior such as daily sleep time, etc.
Logistics and access	Patient’s activation measurements (F17)	How patients are encouraged to continue treatment
	Treatment duration (F18)	Length of therapy and its effect on adherence
	Transport (F19)	Access to healthcare facilities and treatment
	Injection scheduling (F20)	Timing of injections concerning daily routines
	Patient’s satisfaction (F21)	Overall satisfaction with treatment and healthcare
	Tech experience/access (F22)	Availability and familiarity with digital health solutions

F, factors; GHT, growth hormone therapy.

*Details on pain, timing, and caregiver assistance.

^#^Most common causes (forgetfulness and fear of side effects).

^$^Quality of life related to GHD (short stature).

^Sleep patterns/Physical activity (Lifestyle).

### 3.2 First voting round: factor prioritization

**Figure 2:**
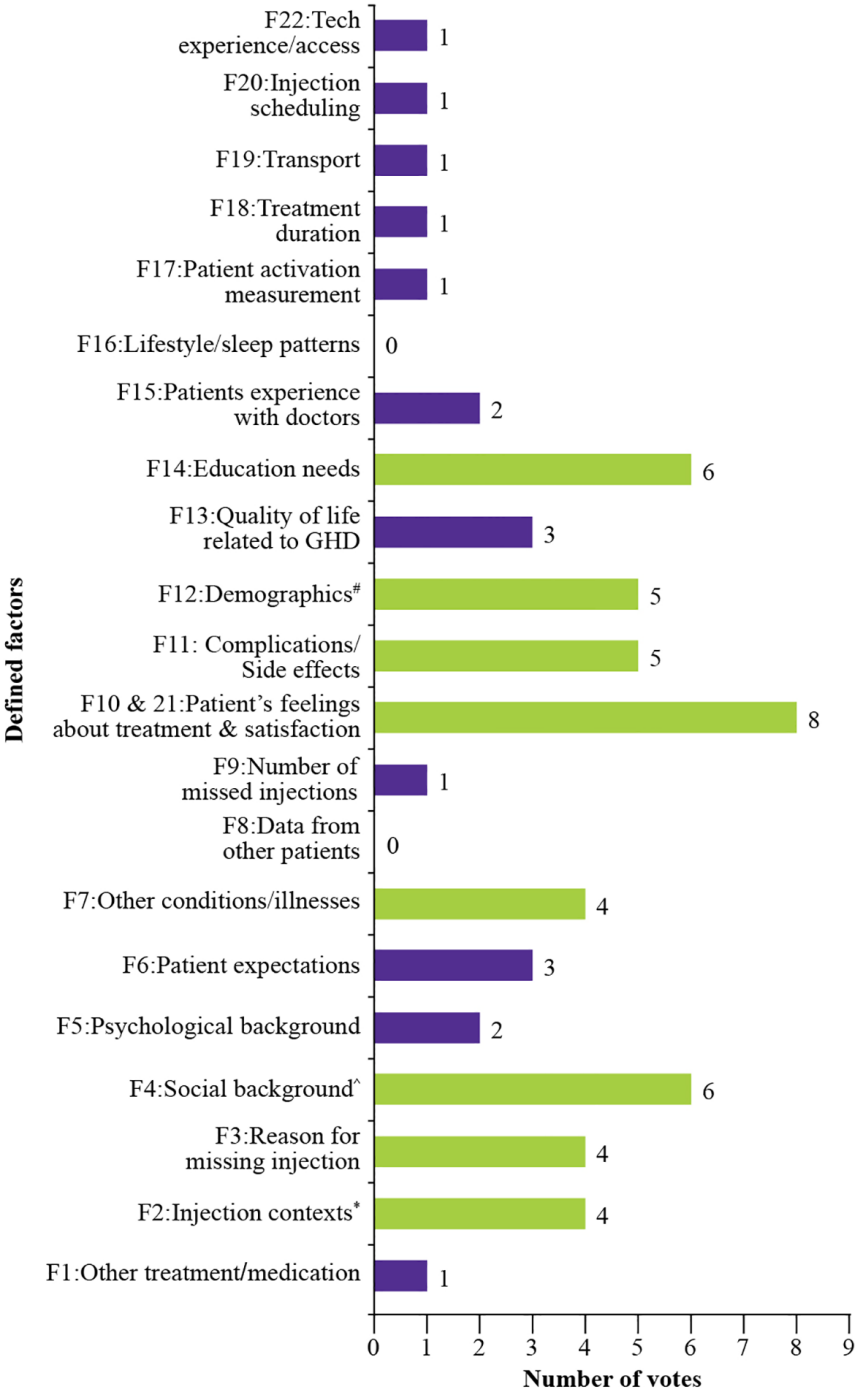
Number of votes per factor in the first ranking round. ^#^Age, income, and familiarity with technology ^*^Comfort, timing, and assistance in administering injections ^^^Family structure and cultural influences F, factor; GHD, growth hormone deficiency.

After an initial list of key factors was completed, experts individually ranked their top five factors based on their perceived importance and real-world impact in clinical settings. This voting process determined the most influential patient-generated health data factors for GHT adherence strategies (**Figure 2**).

Among these, merged factors 10 and 21 (patients’ feelings and satisfaction with treatment) received the highest number of votes. This was followed by factor 4 (social background) and factor 14 (education needs). Factor 12 (demographic data, including age, income level and technology familiarity) was listed in the fourth place in individual votes (tied with factor 11, complications/side effects), but received the highest total votes with 21 points due to a higher weight ranking from the few experts who selected it.

**Figure 3:**
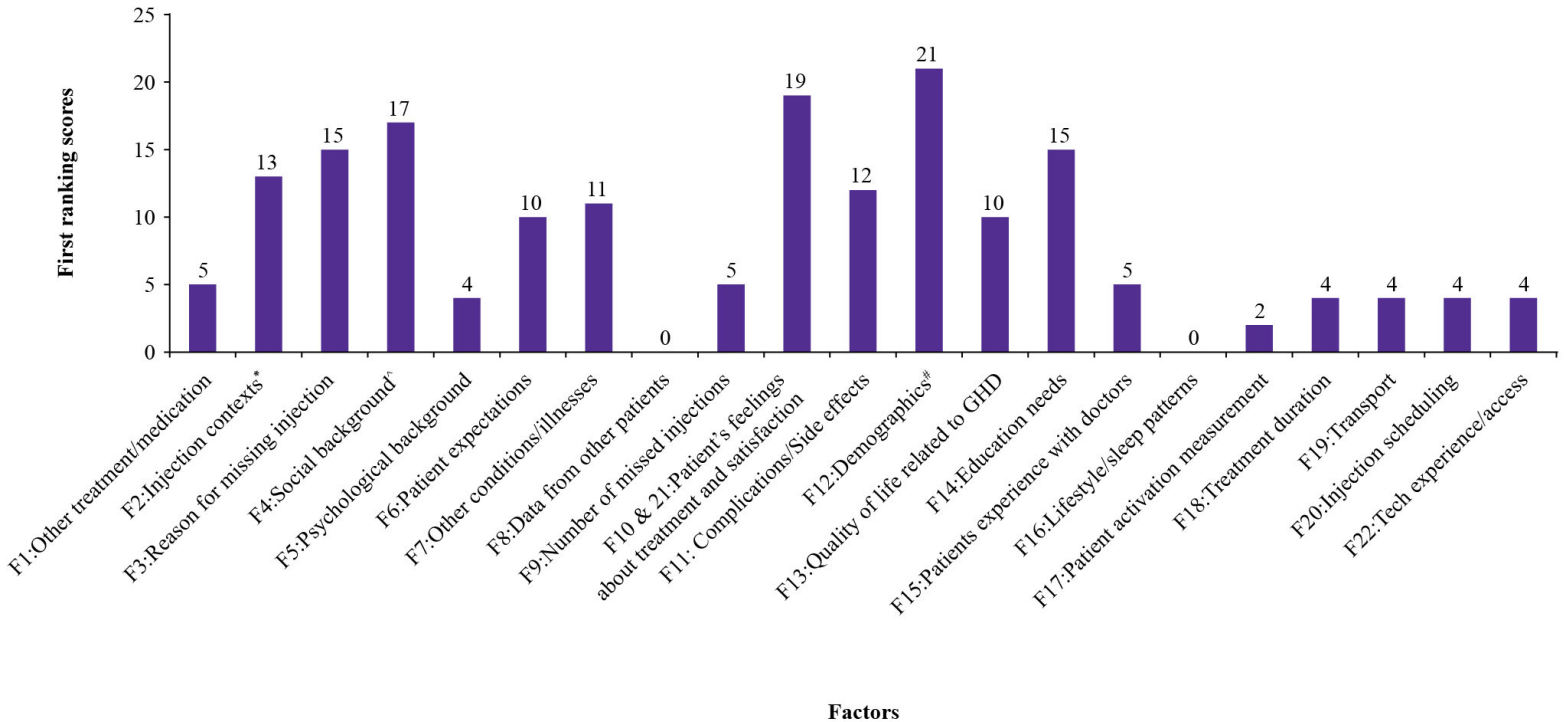
Scores assigned to each factor in the first ranking round. ^#^Age, income, and familiarity with technology ^*^Comfort, timing, and assistance in administering injections ^^^Family structure and cultural influences F, factor; GHD, growth hormone deficiency.

This was followed by the merged factors 10 and 21 (patient’s feelings about treatments and satisfaction, 19 points), factor 4, (social background, 17 points). Factor 3 (reasons for missing injections, 15 points), and factor 14 (educational needs, 15 points), were the fourth most relevant factors. (**Figure 3**).

### 3.3 Second voting round: refining consensus

**Figure 4:**
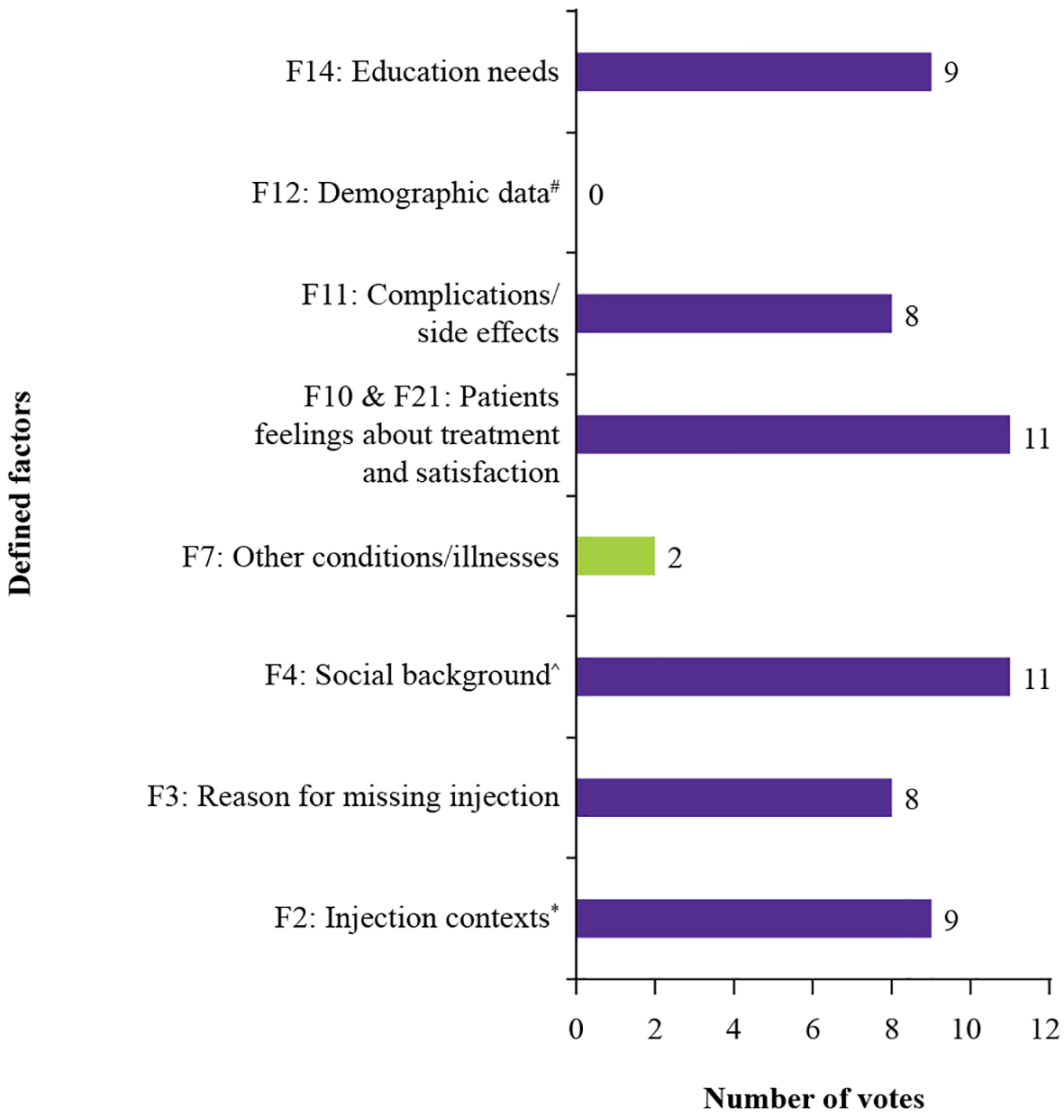
Number of votes per defined factor in the second voting round. ^#^Age, income, and familiarity with technology ^*^Comfort, timing, and assistance in administering injections ^^^Family structure and cultural influences F, factor.

The experts then conducted a second voting round to reassess the top-ranked factors from the first round. Only factors that were selected by at least four experts in the first round were moved forward (**Figure 4**). As per this criterion, eight factors (F2, F3, F4, F7, merged F10 and F21, F11, F12, and F14) were selected for evaluation in the second round of voting (**Figure 4**).

Merged factors 10 and 21(patient’s feelings about treatment and satisfaction), again emerged as the most selected factor in the second voting round, sharing the top position with factor 4 (social background). Factor 12 (demographic data), despite scoring highest in the first ranking round due to a few strong individual rankings, was not selected by any participants in the second round. Only two participants selected factor 7 (other conditions/illnesses).

**Figure 5:**
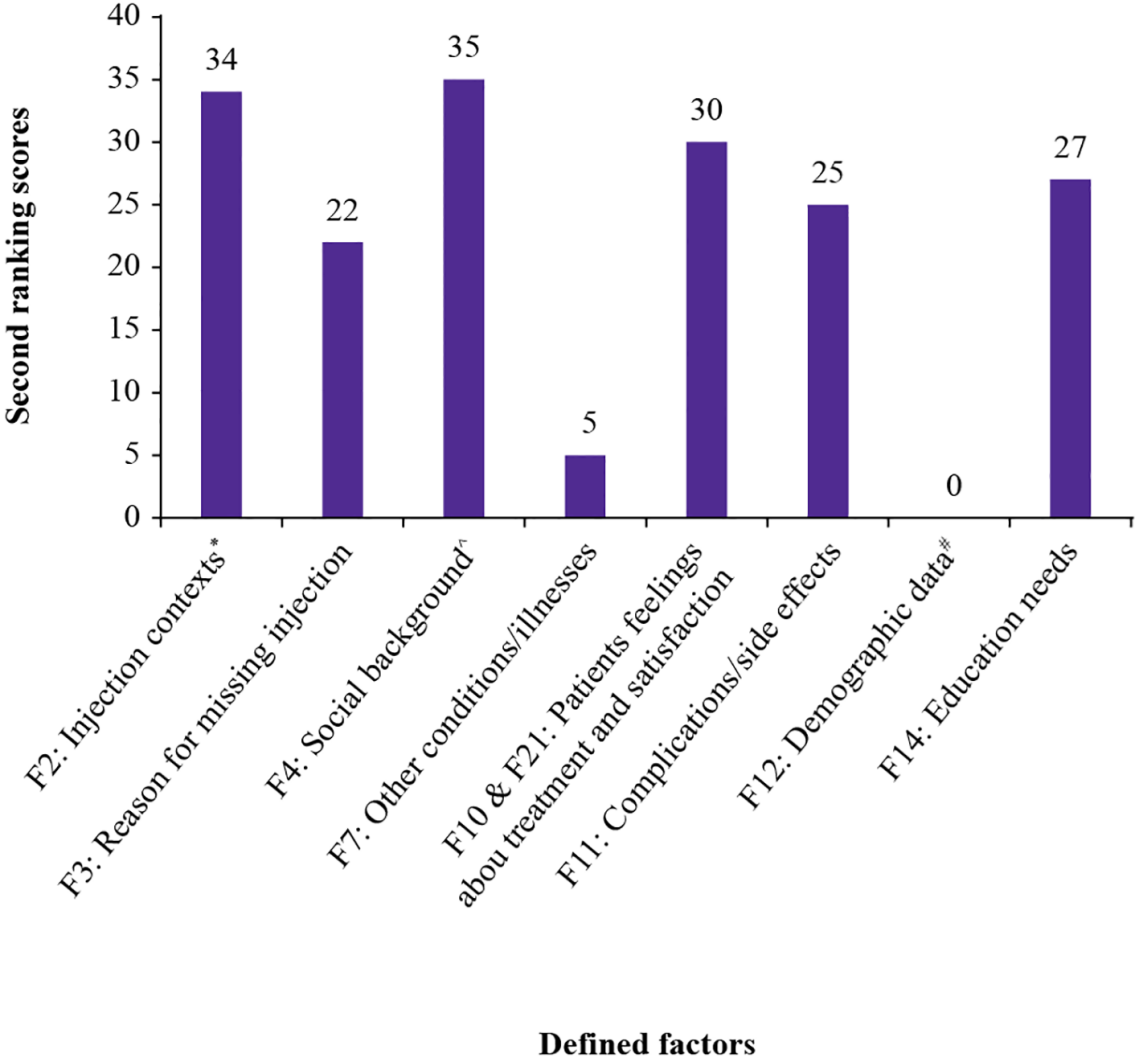
Scores assigned to each factor in the second ranking round. ^#^Age, income, and familiarity with technology ^*^Comfort, timing, and assistance in administering injections ^^^Family structure and cultural influences F, factor.

Following voting, all eight defined factors were scored again, based on the adjusted expert consensus (**Figure 5**). Factor 4, (social background, 35 points), was the most highly scored factor, followed by factor 2 (injection contexts, 34 points), and merged factors 10 and 21 (patient’s feelings about treatment and satisfaction, 30 points).

### 3.4 Final expert discussion

The final discussion focused on how the ranked factors could be integrated into clinical practice to support the management of growth disorders. This question was split into three more specific questions:

- When should these data be available for HCPs?
- In what format should the data be presented to HCPs?
- Which medical team members should receive and analyze these data?

Experts considered multiple options regarding the optimal timing for accessing and analyzing patient-generated health data. The proposed options included:

- Right before the clinic visit.
- One day before a clinic visit.
- One week before a clinic visit.
- One month before a clinic visit.
- After the availability of the laboratory test results (in case of a missed visit).

A key concern that emerged from this discussion was the challenge of time constraints faced by HCPs. Experts emphasized that real-time access to patient-generated health data could be beneficial, but it would increase the workload for clinicians, particularly in busy healthcare settings. Therefore, automated alerts and reminders will be required to facilitate the timely review of patient data. For instance, automated digital alerts aligned with daily routines could remind patients/caregivers to maintain consistency and administer injections at the same time each day, thereby supporting adherence in real-world practice. Experts further highlighted the need for standardized report formats (analogous to continuous glucose monitoring reports for diabetes) to effectively convey patient-generated health data.

Experts agreed that visualizing data in an aggregated, easy-to-interpret format, such as trend graphs or digital tools, would facilitate quicker decision-making. Furthermore, they referenced successful digital health models used in managing chronic diseases, such as diabetes, where structured EHRs have improved adherence monitoring.

The expert panel acknowledged that seamless patient-generated health data integration will require collaboration among physicians, nurses, pharmacists, and patient support programs (PSPs). They emphasized that training and digital literacy among HCPs is essential to ensuring the effective utilization of data.

## 4 Discussion

Growth hormone therapy is essential for managing pediatric growth disorders, yet long-term adherence remains a critical challenge (1, 2). In this study, we conducted a structured expert workshop using the NGT- a validated, multi-step consensus-building approach that is increasingly applied in healthcare research to facilitate expert prioritization and decision making (19, 20). The aim of the workshop was to identify and prioritize the most relevant patient-generated health data factors that could influence adherence to GHT in the GCC region. A total of 22 factors were generated and ranked by experienced pediatric endocrinologists with social background, injection context, and patient feelings about treatment and satisfaction emerging as the most influential factors. These findings emphasize the importance of addressing not only medical aspects but also psychosocial and practical challenges in supporting long-term adherence in pediatric endocrinology (21–23).

The prioritization of social background highlights how deeply family structure, caregiving roles, and broader cultural dynamics affect treatment consistency. In many regions, including the GCC, caregiving and healthcare decisions often involve multiple family members or shared responsibilities, making social determinants particularly relevant to treatment adherence (22, 24). Experts noted that caregiver involvement, emotional support, and financial constraints frequently influence how consistently children follow their injection schedules. This insight is supported by studies showing that family engagement can strongly impact treatment success in pediatric endocrine care (6, 14). Addressing social context in clinical settings may require more than brief consultations, it may involve building caregiver partnerships, using culturally appropriate communication, and designing patient-generated health data systems that reflect real-life routines. Digital health tools such as Easypod^®^ Connect and Growzen^®^ already offer structured dashboards that visualize dosing patterns, which could be adapted to better reflect family involvement and potential adherence barriers (10, 14).

The second most highly prioritized factor was injection context, referring to the timing, comfort, and level of assistance during growth hormone administration. Experts noted that missed doses are often pain, scheduling conflicts, or inconsistent support at home. Environmental or emotional stressors, such as injection anxiety before school or travel, can make treatment burdensome even for motivated families. Prior research is consistent with these insights, noting that discomfort and inconsistent routines are among the most common reasons for missed doses (25). Addressing these issues requires thoughtful education, flexibility, and device-driven support. Smart injection systems, such as Easypod^®^, offer helpful features like real-time tracking, adherence alerts, and personalized dashboards (9, 10). These tools can reveal meaningful trends such as skipped weekend doses or late-night irregularities giving clinicians a more complete picture and enabling more compassionate, targeted counseling. Although the poor responder’s subgroup didn’t yield additional unique factors, its inclusion ensured that this clinically relevant population was addressed within the broader adherence framework. This distinction allows HCPs to differentiate between poor adherence and other biological or psychosocial reasons for suboptimal growth.

The third highest-ranked factor was patients’ feelings about treatment and satisfaction, which includes emotional attitudes, perceived burden, motivation, and expectations. Experts emphasized that even with structured routines and family support, children especially adolescents may experience frustration, fatigue, or disengagement from treatment. This type of “silent” nonadherence is often overlooked unless patients are actively asked or monitored. Prior studies have shown that emotional distress and low perceived value of treatment significantly contribute to missed doses (8, 26). Patient-generated health tools that include brief self-assessments, satisfaction ratings, or mood-tracking features can offer clinicians a deeper window into patients’ lived experiences.

While the value of patient-generated health data is increasingly recognized, our findings highlight several system-level barriers that limits its effective integration. Experts in this study emphasized time constraints, digital literacy challenges, inconsistent data formats and limited interoperability with EHRs, particularly in busy pediatric settings. These limitations reduce the utility of patient-generated health data in day-to-day decision-making. To overcome this, experts recommended using automated alerts, visual summaries, and standardized formats, similar to tools adopted in diabetes care (27). Embedding such features into current EHRs and providing focused training to physicians, nurses, and pharmacists were considered important for sustainable adoption (28–30).

The use of the NGT methodology added valuable structure to this study. It enabled the systematic generation, clarification, and prioritization of ideas, ensuring that all participants contributed equally and that the results reflected shared clinical priorities rather than individual opinions (19, 20). This method was particularly suited to a topic like patient-generated health data, which spans medical, technical, and behavioral domains. The consensus-driven approach also strengthened the validity of the recommendations, helping to generate practical insights that can inform future research and regional implementation strategies.

### 4.1 Limitations

The study is centered on GCC region, which may limit the generalizability of the findings to other regions with differing healthcare infrastructures and digital health adoption patterns. The results are based on an NGT session with selected senior pediatric endocrinologists, the perspectives of other healthcare providers such as nurses, primary care physicians, as well as patients and caregivers were not fully represented. This may potentially limit the comprehensiveness of the findings. For example, it is possible that patients and caregivers may have emphasized on factors such as quality of life, emotional burden, and family dynamics more than clinicians, and these aspects may have been underrepresented.

Despite these limitations, it is worth noting that many of the identified factors including treatment burden, psychosocial barriers, and the integration of digital tools is also observed in pediatric endocrine care globally (11). Therefore, our findings may have broader relevance beyond the GCC context, particularly in healthcare systems with similar delivery settings. Further studies in other regions would help to corroborate the findings from our research.

## 5 Conclusions

Through expert consultation, social background, injection contexts, and patients’ feelings about treatment and satisfaction were identified as key factors influencing the use of patient-generated health data in relation to GHT adherence in the GCC region. GHT adherence is affected by multiple factors, requiring a patient- and caregiver-centered approach, augmented by patient-generated health data, to optimize treatment outcomes. The use of region-specific digital health solutions for GHT can improve adherence by aligning with local clinical practices and fostering collaboration among physicians, nurses, pharmacists, and PSPs. However, challenges such as digital literacy, data standardization, and time constraints must be addressed to facilitate effective adoption of patient-generated health data.

## 6 Future perspectives

Future efforts will focus on enhancing patient- and caregiver-centric support. With real-time injection data and treatment companion apps, it is possible to detect soft signals correlated with suboptimal adherence (e.g., treatment routine, fatigue, teenage independence) and intervene in a timely and direct manner. The insights generated from adherence data on patients can then be shared with the healthcare team to maximize their support to patients and caregivers, thereby improving adherence outcomes, and potentially reducing healthcare costs.

## Data Availability

The original contributions presented in the study are included in the article/supplementary material. Further inquiries can be directed to the corresponding author.
